# Klebsiella pneumoniae sequence type 147: a high-risk clone increasingly associated with plasmids carrying both resistance and virulence elements

**DOI:** 10.1099/jmm.0.001823

**Published:** 2024-04-17

**Authors:** Jane F. Turton, Claire Perry, Kim McGowan, Jack A. Turton, Russell Hope

**Affiliations:** 1 HealthCare Associated Infections, Fungal, Antimicrobial Resistance, Antimicrobial Use and Sepsis Division, UK Health Security Agency, 61, Colindale Avenue, London NW9 5HT, UK; 2 Public Health Microbiology, UK Health Security Agency, 61, Colindale Avenue, London NW9 5HT, UK

**Keywords:** hybrid plasmids, *Klebsiella pneumoniae*, nanopore sequencing, sequence type 147, virulence

## Abstract

**Introduction.** The first hybrid resistance/virulence plasmid, combining elements from virulence plasmids described in hypervirulent types of *Klebsiella pneumoniae* with those from conjugative resistance plasmids, was described in an isolate of sequence type (ST) 147 from 2016. Subsequently, this type has been increasingly associated with these plasmids.

**Hypothesis or gap statement.** The extent of carriage of hybrid virulence/resistance plasmids in nosocomial isolates of *K. pneumoniae* requires further investigation.

**Aim.** To describe the occurrence of virulence/resistance plasmids among isolates of *K. pneumoniae* received by the UK reference laboratory, particularly among representatives of ST147, and to compare their sequences.

**Methodology.** Isolates received by the laboratory during 2022 and the first half of 2023 (*n*=1278) were screened for virulence plasmids by PCR detection of *rmpA*/*rmpA2* and typed by variable-number tandem repeat analysis. Twenty-nine representatives of ST147 (including a single-locus variant) from seven hospital laboratories were subjected to long-read nanopore sequencing using high-accuracy q20 chemistry to provide complete assemblies.

**Results.***rmpA*/*rmpA2* were detected in 110 isolates, of which 59 belonged to hypervirulent K1-ST23, K2-ST86 and K2-ST65/375. Of the remainder, representatives of ST147 formed the largest group, with 22 *rmpA*/*rmpA2*-positive representatives (out of 47 isolates). Representatives were from 19 hospital laboratories, with *rmpA*/*rmpA2*-positive isolates from 10. Nanopore sequencing of 29 representatives of ST147 divided them into those with no virulence plasmid (*n*=12), those with non-New Delhi metallo-β-lactamase (NDM) virulence plasmids (*n*=6) and those carrying *bla*_NDM-5_ (*n*=9) or *bla*_NDM-1_ (*n*=2) virulence plasmids. These plasmids were of IncFIB(pNDM-Mar)/IncHI1B(pNDM-MAR) replicon types. Most of the non-NDM virulence plasmids were highly similar to the originally described KpvST147L_NDM plasmid. Those carrying *bla*_NDM-5_ were highly similar to one another and to previously described plasmids in ST383 and carried an extensive array of resistance genes. Comparison of the fully assembled chromosomes indicated multiple introductions of ST147 in UK hospitals.

**Conclusion.** This study highlights the high proportion of representatives of ST147 that carry IncFIB(pNDM-Mar)/IncHI1B(pNDM-MAR) hybrid resistance virulence plasmids. It is important to be aware of the high probability that representatives of this type carry these plasmids combining resistance and virulence determinants and of the consequent increased risk to patients.

## Introduction

*Klebsiella pneumoniae* is an important nosocomial pathogen increasingly associated with resistance and, at least among some community-acquired types, with hypervirulence [1,3]. The latter, predominantly belonging to sequence types (STs) 23, 86 and 65 of capsular types K1 and K2, carry virulence plasmids containing siderophores, heavy metal resistance genes and regulators of mucoviscosity genes *rmpA*/*rmpA2* [4,5]. These plasmids are generally non-conjugative, but in some cases have combined with conjugative resistance plasmids to become conjugative hybrid resistance/virulence plasmids [6,7]. As a consequence, virulence plasmids are no longer confined to ‘hypervirulent’ types and appear in other types, including ‘high-risk’ clones such as STs 11, 14, 15, 383 and 147, which often carry resistance genes, particularly carbapenemase genes [8,10]. The first hybrid virulence/resistance plasmid was described in an isolate of *K. pneumoniae* ST147 collected in 2016, which also carried *bla*_NDM-1_ in a separate plasmid [11]. Subsequently, isolates carrying both *bla*_NDM-5_ and virulence elements in the same plasmid were described [12,13]. At that time, hybrid resistance/virulence plasmids were remarkable, but they are now becoming common, especially, in our experience, among representatives of ST147. This is in a type already regularly found with at least one carbapenemase, typically NDM and/or OXA-48-like [14,15]; it has even been described with three (KPC-2, NDM-1 and OXA-48) [16].

Other countries have also reported ST147 carrying hybrid virulence/resistance plasmids; for example, there has been a very large outbreak of NDM-1-producing ST147 in Tuscany, Italy with the outbreak strain carrying a large hybrid virulence/resistance plasmid [17,19]. In that case the *bla*_NDM-1_ was in a separate plasmid. Examples have also been reported in Switzerland, Russia and Egypt [20,23]. Here we describe the occurrence of hybrid virulence/resistance plasmids among isolates received by the UK Health Security Agency national reference laboratory for typing and highlight the increasing proportion of representatives of ST147 that carry them. This work was done using long-read nanopore sequencing with q20 chemistry, which provides single-molecule accuracy of at least 99 % [24], allowing complete assembly of isolates and providing a powerful tool for describing these plasmids.

## Methods

### Isolates

Our laboratory provides a typing service to inform cross-infection and outbreak investigations for hospitals in the UK. All isolates of *K. pneumoniae* referred to the laboratory were typed by variable-number tandem repeat (VNTR) analysis at 11 loci. Markers potentially associated with virulence [capsular types K1, K2, K5, K20, K54, K57, *wcaG* (associated with capsular types K1, K16, K54, K58) and *rmpA*/*rmpA2*] were sought by multiplex PCR as described previously [11]. We have previously shown from comparison of VNTR profiles and sequence types mined from whole-genome sequences that the 5,3,5,14,14,2,4,2,5,3,6 VNTR profile corresponds to ST147 [11]. All non-duplicate patient isolates with a profile of 5,3,5,14,14,2,4,2,5,3,6 (*n*=40) and single-locus variants 5,3,5,14,14,2,4,2,5,2,6 (*n*=2), 5,3,5,14,14,2,4,2,5,4,6 (*n*=3) and 5,3,5,14,14,2,4,4,5,3,6 (*n*=2) were assigned as representatives of ST147 (or at least a close variant of it). Isolates selected for nanopore sequencing (Table 1) were chosen to be as representative as possible and to cover the range of isolates seen. They included examples of those likely to be part of an outbreak (related in space and time) and also those from a number of different hospitals so that both epidemiologically related and unrelated isolates were included. Both *rmpA*/*rmpA2*-positive and -negative examples were sequenced.

**Table 1.** Details of representatives of ST147 and ST6796 sequenced, arranged by hospital group and region and summary of virulence and carbapenemase plasmids found. Details of resistance and virulence genes and other plasmids found are provided in Table S1, available in the online version of this article. Hospitals are labelled according to region in England and number within that region (L, London; SE, South East England; WM, West Midlands; YH, Yorkshire and Humber)

Isolate (unique number_hospital_receipt date_ST_carbapenemase gene)	Ward	Isolation site	Virulence resistance plasmid type*	Carbapenemase gene(s) location	Context in which received
KP2_L3_37.21_ST147_NDM5	ITU_L3	Rectal swab	None	*bla*_NDM-5_ in 95.5 kb IncFII plasmid	Single isolate
KP10_L3_11.22_ST147	Neonatal NNU	Neonatal screen	None	None	KP10 and KP11 received together as potentially linked
KP11_L3_11.22_ST147	Neonatal NNU	Neonatal screen	None	None	
KP13_L3_16.22_ST147_NDM1	Surgery ward	Blood	334.1 kb non-NDM	*bla*_NDM-1_ in 53.7 kb IncFIB(pQil) plasmid	KP13 and KP15 from patients in same ward from a further hospital within the group
KP15_L3_19.22_ST147_NDM1	Surgery ward	Rectal swab	Carried *bla*_NDM-1_, but was similar to non-NDM plasmids	*bla*_NDM-1_ in 53.7 kb IncFIB(pQil) plasmid and in virulence plasmid	
KP86_L3_06.23_ST147_NDM14	Acute Medical	Rectal swab	328.5 kb non-NDM†	*bla*_NDM-14_ in 54.1 kb IncFIB(pQil) plasmid	KP86, KP87 and KP88 from three patients on same ward received together as potentially linked
KP87_L3_06.23_ST147_NDM14	Acute Medical	Rectal swab	328.5 kb non-NDM	*bla*_NDM-14_ in 54.1 kb IncFIB(pQil) plasmid	
KP88_L3_06.23_ST147_NDM14	Acute Medical	Rectal swab	328.5 kb non-NDM	*bla*_NDM-14_ in 54.1 kb IncFIB(pQil) plasmid	
KP4_L4_52.21_ST147_OXA181	ITU_L4	Sputum	None	*bla*_OXA-181_ in ≥105 kb IncFII(pKPX1) plasmid	Single isolate
KP28_L6_38.22_ST147_NDM5_OXA232	Elderly Care	Urine	None	*bla*_OXA-232_ in 6.1 kb ColKP3 plasmid; *bla*_NDM-5_ in chromosome	KP28 and KP29 received together as potentially linked
KP29_L6_38.22_ST147_NDM5	Elderly Care	Rectal swab	None	*bla*_NDM-5_ in chromosome	
KP51_L9_46.22_ST147_NDM1_OXA48	Children’s ward	Stool	332.9 kb non-NDM	*bla*_NDM-1_ in 54.1 kb IncFIB(pQil), *bla*_OXA-48_ in 64.4 kb IncL plasmid	Single isolate
KP39_L11_43.22_ST147_NDM5	Not given	Blood	328.5 kb NDM-5‡	*bla*_NDM-5_ in 325.8 kb IncFIB(pNDM-Mar)/IncHI1B(pNDM-MAR) virulence plasmid	Received querying hypervirulence
KP81_L15_03.23_ST147_NDM5	Not given	Blood	352.8 kb NDM-5	*bla*_NDM-5_ in 352.8 kb IncFIB(pNDM-Mar)/IncHI1B(pNDM-MAR) virulence plasmid	KP81 and KP82 isolated within 2 days of each other
KP82_L15_04.23_ST147_NDM5	Not given	Blood	325.8 kb NDM-5	*bla*_NDM-5_ in 325.8 kb IncFIB(pNDM-Mar)/IncHI1B(pNDM-MAR) virulence plasmid	
KP96_L17_12.23_ST6796§_NDM5	Ward A	Rectal swab	335.4 kb NDM-5	*bla*_NDM-5_ in 335.4 kb IncFIB(pNDM-Mar)/IncHI1B(pNDM-MAR) virulence plasmid	KP96 and KP97 received together as potentially linked
KP97_L17_12.23_ST147_NDM5	Ward A	Rectal swab	344.2 kb NDM-5	*bla*_NDM-5_ in 344.2 kb IncFIB(pNDM-Mar)/IncHI1B(pNDM-MAR) virulence plasmid	
KP104_L5_18.23_ST147_NDM5_OXA232	Outpatients	Urine	None	*bla*_NDM-5_ in 156.5 kb IncFII/IncR plasmid, *bla*_OXA-232_ in 6.1 kb plasmid	KP104 and KP124 potentially part of an outbreak; isolated 22 days apart
KP124_L5_21.23_ST147_NDM5	Not given	Urine	343.0 kb NDM-5	*bla*_NDM-5_ in 343.0 kb IncFIB(pNDM-Mar)/IncHI1B(pNDM-MAR) virulence plasmid	
KP99_L14_14.23_ST147_NDM5_OXA48	Ward B	Rectal swab	349.3 kb NDM-5	*bla*_NDM-5_ in 349.3 kb IncHI1B(pNDM-MAR)/IncFIB(pNDM-Mar) virulence plasmid, *bla*_OXA-48_ in 72.2 kb IncL plasmid	KP99 and KP100 received together as potentially linked
KP100_L14_14.23_ST147_NDM5_OXA48	Ward B	Urine	349.3 kb NDM-5	*bla*_NDM-5_ in 349.3 kb IncHI1B(pNDM-MAR)/IncFIB(pNDM-Mar), *bla*_OXA-48_ in ≥67.5 kb IncL plasmid	
KP30_YH2_39.22_ST147_NDM5_OXA181	Ward C	Faeces	None	*bla*_NDM-5_ in 95.9 kb IncFII plasmid, *bla*_OXA-181_ in chromosome	Single isolate
KP63_WM3_49.22_ST147_OXA181	Medical Ward	Rectal swab	None	*bla*_OXA-181_ in 20.4 kb ColKP3 plasmid	Distinct from the two others with which it was received
KP79_WM3_03.23_ST147_OXA181	Ward D	Rectal swab	None	*bla*_OXA181_ in 6.8 kb ColKP3 plasmid	Single isolate
KP102_WM3_17.23_ST147_OXA181	Trauma and Orthopaedic	Rectal swab	None	*bla*_OXA181_ in 12.2 kb ColKP3 plasmid	Single isolate
KP119_WM3_21.23_ST147_NDM1	Ward E	Pooled swabs	352.3 kb NDM-1	352.3 kb IncFIB(pNDM-Mar)/IncHI1B(pNDM-MAR) virulence plasmid	Received with *Enterobacter hormachei* from same patient
KP58_WM2_48.22_ST147_NDM5_OXA48	Trauma	Rectal swab	459.5 kb NDM-5	*bla*_NDM-5_ in 459.9 kb IncFIB(pNDM-Mar)/IncHI1B(pNDM-MAR) plasmid, *bla*_OXA-48_ in 68.9 kb IncL plasmid	Received in a large set; only one of this type
KP103_WM5_18.23_ST147_OXA48	Not given	Bloodculture	None	*bla*_OXA-48_ in 78.6 kb IncL plasmid	Received with other OXA-48 isolates of different types/species querying if related
KP101_SE2_14.23_ST147_NDM5_OXA232	Haematology ward	Faeces	334.3 kb non-NDM	*bla*_NDM-5_ in 53.4 kb IncFIB(pQil), *bla*_OXA-232_ in 12.3 kb ColKP3§ plasmid	Single isolate

a *All the virulence resistance plasmids were of IncFIB(pNDM-Mar)/IncHI1B(pNDM-MAR) replicon type and carried the aerobactin cluster (*iucABCD*, *iutA*), *rmpA*/*rmpA2*, tellurite resistance genes *terABCDEWXYZ*, *shiF*, lysozyme inhibitor and *cobW* genes. They are summariszed as either non-NDM, NDM-1 or NDM-5 plasmids.

b† nNon-NDM virulence plasmids were similar to KpvST147L_NDM and all carried *armA*, *msr(E), mph(E*) and *sul2* in addition to the virulence genes, and variably carried *aph(3')-Ia*, *mph(A*), *qnrS1*, *sul1* and *dfrA5*.

c ‡NDM-5 virulence plasmids all carried *bla*_NDM-5_, *aadA1*, *aph(3')-VI*, *bla*_CTX-M-15_, *bla*_TEM-1B/C_, *bla*_OXA-9_, truncated *catA1* and *qnrS1* in addition to the virulence genes and variably carried *aph(3')-Ia*, *aac(6')-Ib*, *aac(6')-Ib3, mph(A*), *sul1*, *sul2* and *dfrA5*.

d §ST6796 is a single-locus variant (SLV) of ST147.

e¶ This plasmid consists of two copies of a 6.1 kb sequence.

NNU, neonatal unit; ITU intensive therapy unit.

Isolates subjected to nanopore sequencing were labelled by a unique number (prefixed by KP) followed by a hospital code, the week and year of receipt by the laboratory, their sequence type and the carbapenemase genes that they carried (e.g. KP2_L3_37.21_ST147_NDM5). Hospitals were labelled according to the region in England from which they were received and the number within that region (L, London; SE, South East England; WM, West Midlands; YH, Yorkshire and Humber).

This study was part of a larger one that involved sequencing a further 98 isolates of *K. pneumoniae*. Where relevant, isolates belonging to other sequence types (ST395, ST2096 and ST1558) that carried virulence plasmids are mentioned; these were sequenced in exactly the same way as those belonging to ST147. These sequences are also available under project PRJNA1010831.

### Nanopore sequencing

Cultures from single-colony picks of 28 representatives of ST147 and 1 single-locus variant (SLV) (ST6796) collected between late 2021 and the end of June 2023 were sequenced on R10.4.1 flow cells on a minION Mk1C (or gridION for run q20run8) following library preparation using the rapid barcoding kit SQK-RBK114.24. Library preparation was carried out exactly as described in the protocol provided by Oxford Nanopore Technologies, with concentration of the pooled barcoded libraries using ‘clumping buffer’ [25] as described previously [26] (q20runs 2–7) or Ampure beads (q20runs 8–16) provided in the kit. Basecalling was done locally using the high-accuracy basecalling option with barcode trimming. MinKNOW versions were 22.10.7 for q20 run 2 (rrp9), 22.12.5 (bionic) for q20 runs 5, 7, 8, 10, 13 and 14 and 23.04.5 (bionic) for q20 runs 15 and 16. For runs 2–14 a speed of 260 bp s^−1^ was used; with minKNOW version 23.04.5 (bionic) (q20runs 15 and 16) only a 400 bp s^−1^ option was available.

### Assembly

Sequences were assembled using flye 2.9.1-b1780 [27] and medaka 1.7.2, a neural network correction tool provided by Oxford Nanopore technologies (https://github.com/nanoporetech/medaka). Resistance genes and plasmid replicon types were sought using ResFinder 4.3.3 and PlasmidFinder 2.1, respectively, on the Center for Genomic Epidemiology website (https://cge.food.dtu.dk/) [28,29]. Multilocus sequence typing (MLST) types were checked using MLST 2.0 on that website [30]. Virulence and heavy metal resistance elements were detected by blast comparisons with the relevant sequences all included in a fasta file. Capsular (K)/K-locus (KL) types and aerobactin sequence types were sought from the Pasteur *Klebsiella pneumoniae* bigsdb database (https://bigsdb.pasteur.fr/klebsiella/).

Assembled sequences were submitted to GenBank under project PRJNA1010831 and accession numbers CP137420–CP137423 (KP10_L3_11.22_ST147), CP137403–CP137405 (KP11_L3_11.22_ST147), CP137386–CP137390 (KP39_L11_43.22_ST147_NDM5), CP137396–CP137402 (KP119_WM3_21.23_ST147_NDM1), CP137379–CP137385 (KP51_L9_46.22_ST147_NDM1_OXA48), CP137375–CP137378 (KP58_WM2_48.22_ST147_NDM5_OXA48), CP137369–CP137374 (KP81_L15_03.23_ST147_NDM5), CP137431–CP137435 (KP82_L15_04.23_ST147_NDM5), CP137362–CP137368 (KP86_L3_06.23_ST147_NDM14), CP137424–CP137430 (KP88_L3_06.23_ST147_NDM14), CP137357–CP137361 (KP97_L17_12.23_ST147_NDM5), CP137351–CP137356 (KP99_L14_14.23_ST147_NDM5_OXA48), CP137413–CP137419 (KP101_SE2_14.23_ST147_NDM5_OXA232), CP137406–CP137412 (KP102_WM3_17.23_ST147_OXA181), CP137391–CP137395 (KP124_L5_21.23_ST147_NDM5) and CP138472–CP138477 (KP96_L17_12.23_ST6796_NDM5).

### Chromosome comparisons

Fully assembled chromosomes were compared by split kmer analysis (SKA) with the tool ‘SKA’ (https://github.com/bacpop/ska.rust, version 2 release v0.3.2) developed by Harris [31] with a kmer size of 31. Alignment was created against the chromosome of KP96_L17_12.23_ST6796_NDM5 (identified as having the lowest median mash distance from the others) using SKA map with ‘ambig mask’ option. This alignment was used to create a tree with Gubbins [32] v3.3.1 (which corrects for recombination) using default settings and 1000 bootstraps.

## Results and discussion

### Isolates carrying virulence plasmids

During 2022 and the first half of 2023, 1278 non-duplicate patient isolates of *K. pneumoniae* were referred to the laboratory for typing from 97 hospital laboratories to inform cross-infection investigations and/or to detect ‘hypervirulence’ elements. The majority (49 %) were from screening (from rectal swabs, faeces, nasal and pooled swabs), but they also included those from blood (22 %), tissue, fluids, pus, abscesses (3 %), urine (12 %), wounds (3 %) and sputum and other respiratory samples (6 %). Regulators of mucoviscosty phenotype genes *rmpA*/*rmpA2* (indicative of a virulence plasmid) were detected in 110 of these (110/1278). Of these, 49 belonged to hypervirulent K1 clonal group 23, 6 belonged to hypervirulent K2-ST86 of VNTR type (5,8/IS,6,1,1,2,3,2,3,6/7), 4 to hypervirulent K2-ST65/375, 4 to K2 isolates of VNTR profile 5,-,0,1,1,1,1,4,3,2,4, 2 to K2-CG380 (6,4,-10,-,2,2,3,3,3,3), 4 to hypervirulent K54-CG29 and 2 to K57 isolates of VNTR type 5,2,2,20,1,2,4,4,4,2,4. Of the remainder, it was striking that representatives of ST147 formed the biggest group, with 22 *rmpA*/*rmpA2*-positive representatives (out of 47 isolates) (47 %), followed by ST14/2096 (5 out of 48 representatives). Other high-risk clones were not well represented among *rmpA*/*rmpA2* positive isolates – there were just two isolates of ST101 and one of ST383. Four (of 17) representatives of ST395 carried *rmpA*/*rmpA2*. Others have also recently described representatives of ST395 carrying hypervirulence plasmids [33]. Representatives of ST147 were from 19 hospital laboratories, with *rmpA*/*rmpA2*-positive isolates received from 10 of them.

### Nanopore-sequenced isolates of ST147 and SLV ST6796

Since isolates belonging to ST147 were second only to those of K1-ST23 in being the most common type carrying virulence plasmids (evidenced by detection of *rmpA*/*rmpA2*) among referrals to our laboratory, these were studied further by sequencing 29 representatives, of which 27 were received in the study period above and 2 were received in 2021 (Table 1 and S1). Twelve of the 29 isolates sequenced (from 7 hospital laboratories) of ST147 and ST6796 (SLV of ST147) did not carry a virulence plasmid, although 1 had a 200 kb IncFIB(pNDM-Mar)/ IncHI1B(pNDM-MAR) plasmid that included the tellurite resistance genes *terABCDEWXYZ*, but none of the other elements associated with virulence plasmids [such as the aerobactin cluster (*iucABCD*, *iutA*) and *rmpA*/*rmpA2*] (KP102_WM3_17.23_ST147_OXA181; CP137411) (Table S1). All but two (both from neonates) carried at least one carbapenemase gene; *bla*_OXA-48_ (*n*=1), *bla*_OXA-181_ (*n*=4), *bla*_NDM-5_ (*n*=2) or both *bla*_OXA-181_ and *bla*_NDM-5_ (*n*=1); two isolates carried the *bla*_OXA-232_ and *bla*_NDM-5_ combination (in separate plasmids). Interestingly the plasmids carrying *bla*_NDM-5_ in these isolates that did not carry virulence plasmids were IncF plasmids also carrying *bla*_TEM-1B_ (encoding resistance to beta-lactams), *dfrA12*, *sul1* (folate pathway antagonists), *erm(B*), *mph(A*) (encoding resistance to macrolides), *aadA2* and *rmtB* (encoding resistance to aminoglycosides), mirroring a similar situation that we have observed in *Escherichia coli* [34]. Even those from the neonates (KP10_L3_11.22_ST147 and KP11_L3_11.22_ST147) carried a significant complement of resistance genes, including multiple beta lactam (e.g. *bla*_CTX-M-15_, *bla*_OXA-1_, *bla*_TEM-1B_), aminoglycoside [*aadA1*, *aph(6)-Id*, *aph(3″)-Ib*, *aac(3)-IIa*, *aac(6')-Ib-cr*], folate pathway antagonists (*sul1*, *sul2*, *dfrA14*, *dfrA15*), macrolide [*erm(B*), *mph(A*)], tetracycline [*tet(D*)], quinolone (*qnrB2*) and amphenicol (*catA2*) resistance genes, in addition to the *fosA* (encoding resistance to fosfomycin), *oqxAB* (encoding resistance to folate pathway antagonists/quinolones/quaternary ammonium compounds/amphenicols) and *bla*_SHV-11/67_ (encoding resistance to beta-lactams; nearest alleles given) genes found in all the isolates.

### Hybrid virulence resistance plasmids

**Fig. 1.**
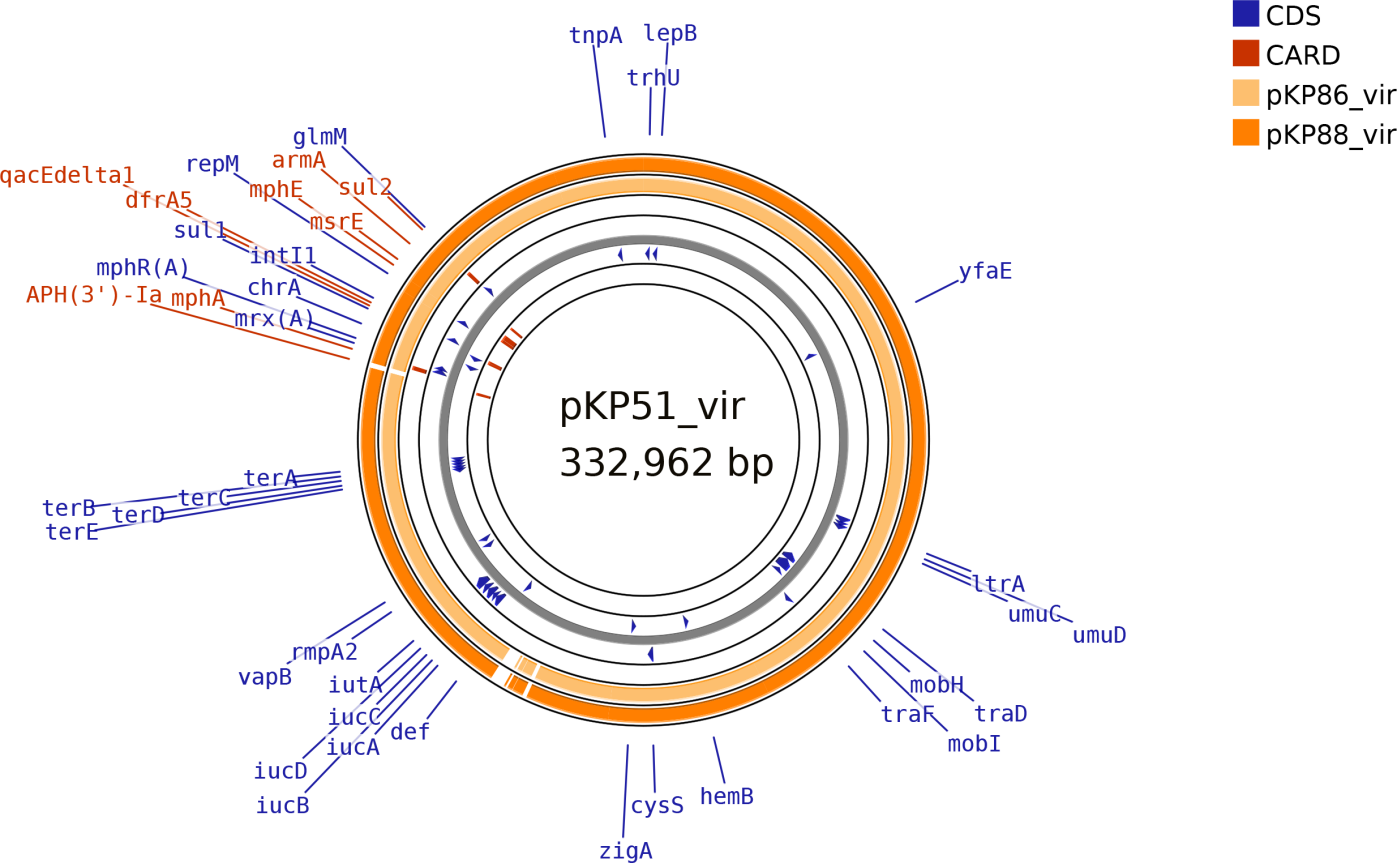
blast comparison of examples of hybrid virulence plasmids not containing NDM from ST147 isolates. Plasmids from KP86_L3_06.23_ST147_NDM14 (pKP86_vir, CP137363) and KP88_L3_06.23_ST147_NDM14 (pKP88_vir, CP137424) are compared with that of KP51_L9_46.22_ST147_NDM1_OXA48 (pKP51_vir, CP137382). Coloured areas represent homology with pKP51_vir. Only those coding sequences (CDSs) that are labelled are shown. Purple labels are from the GenBank annotation of CP137382, while red labels are from comparison with the Comprehensive Antibiotic Resistance Database (CARD). The figure was generated using Proksee (https://proksee.ca/) [37].

The remaining isolates (*n*=17) all carried plasmids containing the aerobactin cluster (*iucABCD*, *iutA*), *rmpA*/*rmpA2*, tellurite resistance genes *terABCDEWXYZ*, *shiF*, lysozyme inhibitor and *cobW* genes (typical of virulence plasmids in hypervirulent types) and multiple antibiotic resistance genes. These included four of the five isolates from blood. These hybrid virulence/resistance plasmids could be divided into those not carrying an NDM gene (although often *bla*_NDM_ was carried in a separate plasmid) (*n*=6), those carrying *bla*_NDM-5_ (*n*=9) and those carrying *bla*_NDM-1_ (*n*=2) (Tables 1 and S1). All these hybrid virulence plasmids were of IncFIB(pNDM-Mar)/IncHI1B(pNDM-MAR) replicon types. Those not carrying a *bla*_NDM_ gene all carried *armA* (encoding resistance to aminoglycosides), *sul2* (folate pathway antagonist), *msr(E*) and *mph(E*) (encoding resistance to macrolides) in addition to the virulence genes, with some also carrying *aph(3')-Ia* (encoding aminoglycoside resistance) (2/6), *mph(A*) (encoding resistance to macrolides) (5/6), *sul1* (5/6), *qnrS1* (encoding ciprofloxacin resistance) (1/6) and *dfrA5* (encoding trimethoprim resistance) (5/6) in addition (Fig. 1), and were similar to KpvST147L_NDM (CM007852), which was the first hybrid virulence resistance plasmid we noted in an isolate of ST147 from 2016 (e.g. KpvST147L_NDM shares 100 % coverage and 99.98 % identity with pKP51_vir). It is also similar to ones we have found from representatives of ST395 from hospitals NW3 and WM2 (e.g. CP133748) and of ST2096 from hospital NE2 (CP140295) received during 2022 and early 2023. One of the *bla*_NDM-1_ virulence plasmids (in isolate KP15_L3_19.22_ST147_NDM1) appeared to carry this gene in both the virulence plasmid and in a separate IncFIB(pQil) plasmid, but the virulence plasmid was otherwise similar to those in the non-NDM virulence plasmids.

The virulence plasmids carrying *bla*_NDM-5_ were highly similar to one another, all carrying *aadA1*, *aph(3')-VI*, *bla*_CTX-M-15_, *bla*_TEM-1B/C_, *bla*_OXA-9_, truncated *catA1* and *qnrS1*, and most carrying *mph(A*) (7/19), *sul1* (8/9), *dfrA5* (7/9) and *aac(6')-Ib* (7/9) or *aac(6')-Ib3* (2/9) in addition; less commonly *sul2* (4/9) and *aph(3')-Ia* (3/9) were present. The further plasmid carrying *bla*_NDM-1_ had a slightly different complement of resistance genes carrying *aph(3')-VI*, *aac(6')-Ib-cr*, *bla*_CTX-M-15_, *bla*_OXA-1_, *sul1*, *qnrS1*, *ARR-3* (encoding rifampicin resistance) and *catB3*. The isolate carrying this *bla*_NDM-1_ virulence/resistance plasmid (KP119_WM3_21.23_ST147_NDM1) differed from the others in belonging to capsular type K20; those carrying the *bla*_NDM-5_ virulence/resistance plasmids all belonged to a KL64 type.

**Fig. 2.**
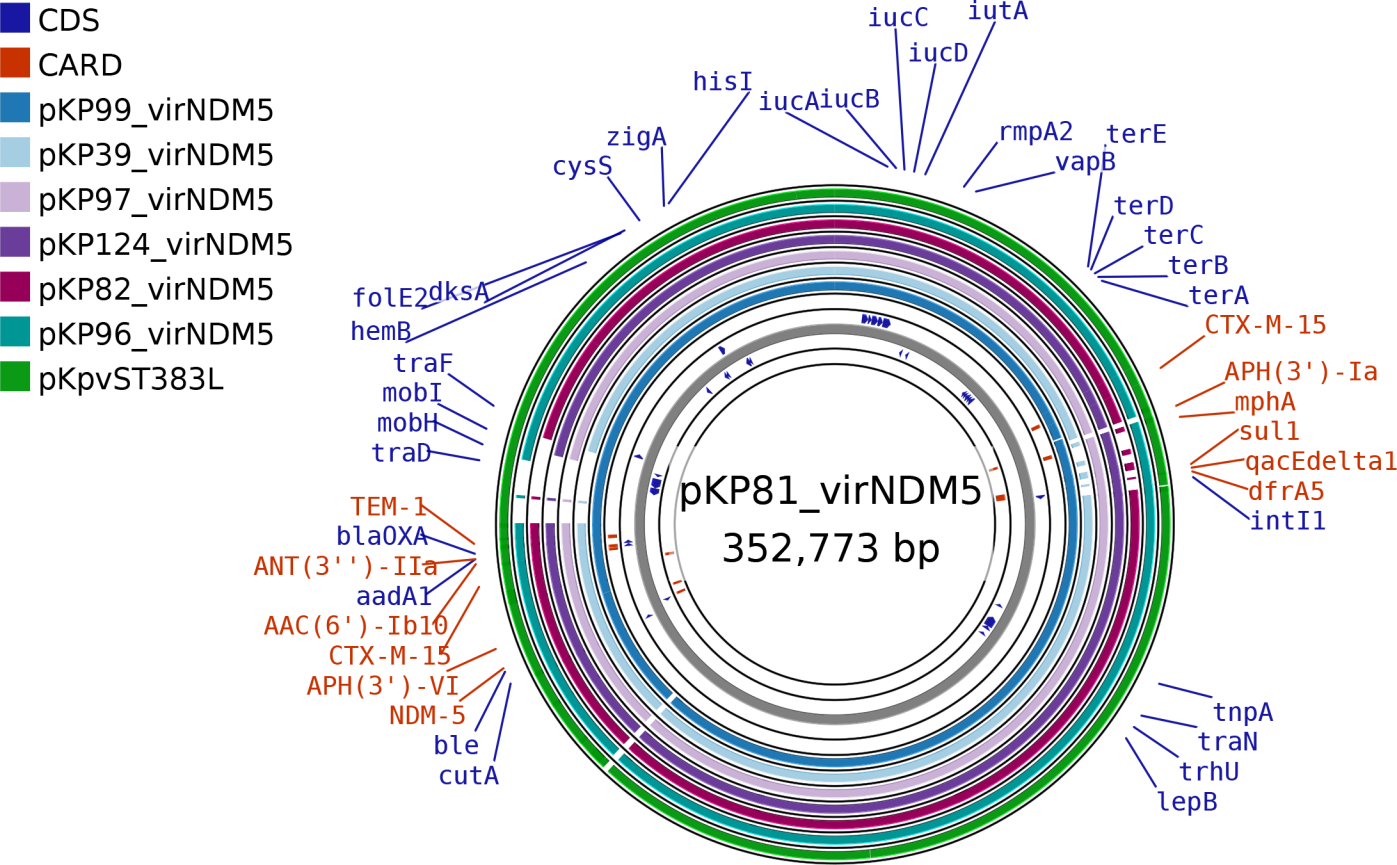
blast comparison of the *bla*_NDM-5_ hybrid virulence plasmids from ST147 isolates KP99_L14_14.23_ST147_NDM5_OXA48 (pKP99_virNDM5, CP137352), KP39_L11_43.22_ST147_NDM5 (pKP39_virNDM5, CP137386), KP97_L17_12.23_ST147_NDM5 (pKP97_virNDM5, CP137360), KP124_L5_21.23_ST147_NDM5 (pKP124_virNDM5, CP137392), KP82_L15_04.23_ST147_NDM5 (pKP82_virNDM5, CP137435), KP96_L17_12.23_ST6796_NDM5 (pKP96_virNDM5, CP138475) and an isolate belonging to ST383 described previously (pKpvST383L, CP034201) with KP81_L15_03.23_ST147_NDM5 (pKP81_virNDM5, CP137372), showing the high similarity between them, despite their having been isolated from multiple hospitals. Coloured regions represent areas of homology with pKP81_virNDM5. Only those coding sequences (CDSs) that are labelled are shown. Purple labels are from the GenBank annotation of CP137392, while red labels are from comparison with the CARD database. The figure was generated using Proksee (https://proksee.ca/) [37].

Many of the *bla*_NDM-5_ hybrid virulence/resistance plasmids [e.g. those from KP39_L11_43.22_ST147_NDM5 (CP137386), KP58_WM2_48.22_ST147_NDM5_OXA48 (CP137375), KP81_L15_03.23_ST147_NDM5 (CP137372), KP82_L15_04.23_ST147_NDM5 (CP137435), KP97_L17_12.23_ST147_NDM5 (CP137360), KP99_L14_14.23_ST147_NDM5_OXA48 (CP137352) and KP124_L5_21.23_ST147_NDM5 (CP137392)] were highly similar to that we have previously described in an isolate of ST383 (CP034201) (Fig. 2). There was also high similarity with plasmid pFQ61_ST383_NDM-5 (CP091814) from an isolate of ST383 from Spain and with those from 2022CK-00752 and 2022CK-00768 from the USA (CP117746.1 and CP117741.1), both belonging to ST147 and all also carrying *bla*_NDM-5_. We have also observed a similar plasmid in an isolate of ST1558 from hospital WM1 (CP141546) isolated in early 2023.

### Chromosome comparison

**Fig. 3.**
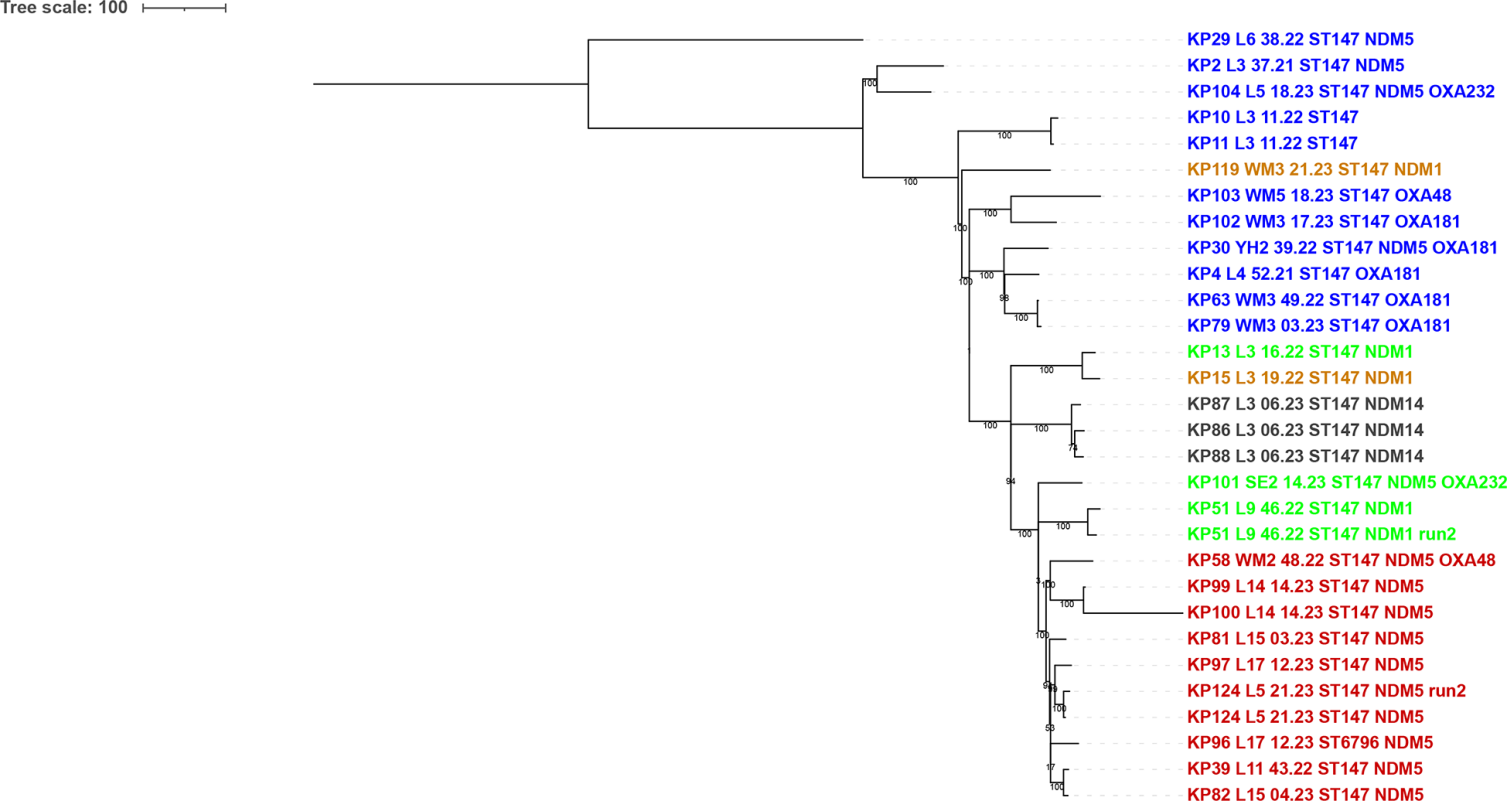
Comparison of the fully assembled chromosomes from isolates in this study. Comparison was by split kmer analysis (SKA) [31]. Chromosomes were aligned against the chromosome of KP96_L17_12.23_ST6796_NDM5 and a tree created with Gubbins [32]. The tree was rooted on the KP29_L6_38.22_ST147_NDM5 outlier. Isolates are labelled with a unique KP number followed by a hospital code, receipt date (week.year), sequence type (ST) and any carbapenemase genes carried (in any contig). Isolates are coloured according to whether they had no hybrid virulence plasmid (in blue), a non-NDM hybrid virulence plasmid (in grey and in green), those with a hybrid virulence resistance plasmid carrying *bla*_NDM-1_ (highlighted in gold) and those with a hybrid virulence resistance plasmid carrying *bla*_NDM-5_ (highlighted in crimson), as in Table S1. The comparison is of the closed chromosome contigs alone. Bootstrap values shown are from 1000 replicates. Results from two separate runs are included on two isolates (KP51_L9_46.22_ST147_NDM1_OXA48 and KP124_L5_21.23_ST147_NDM5).

Since the sequences for most of these isolates (28/29) were fully assembled into closed chromosome and plasmid contigs, this afforded the possibility of comparing the complete chromosome sequences in the absence of the plasmid sequences (Fig. 3). The comparison included chromosome assemblies from two different runs carried out on the same extract for one of the isolates (KP124_L5_21.23_ST147_NDM5) and from runs on two different extracts (from different picks) for another of the isolates (KP51_L9_46.22_ST147_NDM1_OXA48) to assess the potential variation expected for an isolate. These different runs clustered most closely with the other run of the pair. The comparison revealed that the chromosomes of the two isolates from neonates on the same ward (KP10_L3_11.22_ST147 and KP11_L3_11.22_ST147) clustered very closely together, as did the chromosomes of those that also carried *bla*_NDM-14_ plasmids (KP86_L3_06.23_ST147_NDM14, KP87_L3_06.23_ST147_NDM14 and KP88_L3_06.23_ST147_NDM14), also from that hospital group but clearly distinct from the neonatal isolates. The chromosomes of all of the isolates carrying *bla*_NDM-5_ virulence plasmids were all in the same broad clade. These isolates did not cluster according to hospital, showing that isolates of this widely found high-risk clone from the same hospital are not necessarily closely linked. However, the chromosomes of KP13_L3_16.22_ST147_NDM1 and KP15_L3_19.22_ST147_NDM1 from the same hospital (L3) did cluster with one another. The chromosomes of the isolates that did not carry virulence plasmids clustered apart from those that did, but mostly were not closely related. However, in addition to the aforementioned neonatal isolate cluster, two isolates from hospital WM3 clustered together (KP63_WM3_49.22_ST147_OXA181 and KP79_WM3_03.23_ST147_OXA181). Interestingly, the chromosome of KP29_L6_38.22_ST147_NDM5 was an outlier from the rest; this isolate differed from the others in that the assembly put the *bla*_NDM_ gene (*bla*_NDM-5_ in this case) in the chromosome rather than in a plasmid; this was the case in two separate runs done on this isolate.

While there was clear evidence for limited transmission between patients among the set, fortunately, we did not observe the rapid and extensive spread observed by Martin *et al*. and others in the outbreaks of *K. pneumoniae* ST147 in Italy [18,35]. Nevertheless, that these isolates are widely found among UK hospitals and that they often carry conjugative hybrid resistance/virulence plasmids is extremely worrying. Moreover, we have observed a clear link between the *bla*_NDM-5_ hybrid plasmids found in isolates of ST147 and those in ST383, both from the UK and from Spain, highlighting their capacity to move to other high-risk clones.

While it is unlikely that these isolates that carried hybrid virulence/resistance plasmids display the full virulence characteristics associated with the typical hypervirulent types such as K1-ST23 and K2-ST86, 5 of the 29 isolates sequenced were from blood, indicating invasive disease. One of these (KP39_L11_43.22_ST147_NDM5) was received as a query hypervirulent strain, suggesting a virulent phenotype. All but one (KP119_WM3_21.23_ST147_NDM1) also carried genes encoding yersiniabactin in the chromosome, also regarded as a virulence factor, but that is not unusual among currently circulating clinical isolates of * K. pneumoniae*. Studies on isolates from the outbreak in Tuscany showed that they did not exhibit the full hypervirulent phenotype when tested in the *Galleria mellonella* infection model and in a subcutaneous model of infection in immunocompetent CD1 mice. They showed variably enhanced serum resistance in serum bactericidal assays [18,19], depending on *pal*, *csrD* and *ramR* genes encoding surface components in the chromosome, with inactivation of CsrD associated with increased capsule production and thickness and a consequent increased serum fitness.

In common with other studies, our isolates were divided into distinct K/KL types. Six of the 29 isolates sequenced, all lacking virulence plasmids, belonged to the KL10 type, described as being predominantly found in Asian countries and associated with NDM and OXA-48-like carbapenemases [15]. However, most of the isolates (22/29), including all those carrying *bla*_NDM-5_ virulence plasmids belonged to the KL64 type, associated with Europe [10]. One isolate belonged to the K20 capsular type; this seems to be more rarely found but has been described among isolates from Switzerland [20] and Russia [21].

The two isolates from neonates described here were unusual in that they carried no carbapenemase genes, unlike almost every other isolate of this type received by the UK reference laboratory. All the isolates, even these relatively susceptible ones, carried an extensive array of resistance genes. For example KP99_L14_14.23_ST147_NDM5_OXA48 carried *aph(3')-Ia*, *aadA1*, *aph(3')-VI*, *aac(6')-Ib*, *bla*_CTX-M-15_, *bla*_TEM-1B/C_, *bla*_OXA-9_, *mph(A*), truncated *catA1*, *qnrS1*, *sul1* and *dfrA5*, in addition to *bla*_NDM-5_ in the NDM-5 hybrid virulence/resistance plasmid, *aph(6)-Id*, *aph(3'')-Ib*, *aph(3')-VIb* and *bla*_CTX-M-14b_, in addition to *bla*_OXA-48_ in the OXA-48 plasmid, and *rmtF*, *aac(6')-Ib-Hangzhou*, *ARR-2*, *fosA*, *oqxAB* and *bla*_SHV-11/67_ in other plasmids and the chromosome (nearest alleles given where there is not an exact match). We are not aware of colistin resistance among the representatives described here but note the experience in Tuscany, where a colistin and tigecycline-resistant variant evolved during the outbreak as a result of mutations in RamR and MgrB proteins, resulting in premature stop codons and associated with tigecycline and colistin resistance, respectively [36]. We have previously reported colistin resistance in representatives of ST147 that was not associated with *mcr* genes (MIC 8 mg l^−1^) [11], as have others [16,18], with Di Pilato *et al.* also noting mutations in chromosomal genes associated with colistin resistance (*pmrA, crrB, mgrB*) among representatives of ST147. Phenotypic susceptibility testing on isolates of ST147 has been reported by many authors and reflects the substantial complement of resistance genes that they carry [11,16, 18, 19, 23].

Isolates received by the national reference laboratory will not have been without bias; the mere fact that they were received for typing means that they will have been biased towards incidents of suspected or actual cross-infection. Some hospitals choose to send isolates while others do not. Nevertheless, the results can identify trends, and the finding of virulence plasmids in representatives of ST147 from multiple hospital laboratories (19) and regions (4) implies a widespread issue.

In conclusion, this study has shown that hybrid virulence resistance plasmids in *K. pneumoniae* were strongly associated with ST147 among referrals to the UK reference laboratory during 2022/3. In terms of numbers, ST147 was second only to K1-ST23 (the archetypal hypervirulent type) in being the type most associated with virulence plasmids among isolates referred to us (there were 49 isolates of K1-ST23 and 22 of ST147 carrying virulence plasmids on the basis of *rmpA*/*rmpA2* screening). In ST147, these are conjugative plasmids combining virulence and resistance elements. Almost half of representatives of this type carried these plasmids. Most worryingly, a hybrid plasmid carrying *bla*_NDM-5_ was widely found among representatives and has also been described in representatives of ST383 and of ST1558, highlighting a wide distribution and the potential for spread between types. These observations show that ST147 should be considered a high-risk clone for carriage of these plasmids. Description of these plasmids was greatly facilitated by nanopore sequencing, which enabled complete assemblies of both the chromosomes and plasmids of these isolates.

## supplementary material

10.1099/jmm.0.001823 Uncited Table S1.

## Abbreviations

AbST: aerobactin sequence type
CARD: comprehensive antibiotic resistance database
CDS: coding sequence
ITU: intensive therapy unit
K/KL: Capsular and K-locus types
NDM: New Delhi metallo-β-lactamase
NNU: neonatal unit
SKA: split kmer analysis
SLV: single-locus variant
ST: sequence type
VNTR: variable-number tandem repeat analysis
